# Human Pose Estimation from Monocular Images: A Comprehensive Survey

**DOI:** 10.3390/s16121966

**Published:** 2016-11-25

**Authors:** Wenjuan Gong, Xuena Zhang, Jordi Gonzàlez, Andrews Sobral, Thierry Bouwmans, Changhe Tu, El-hadi Zahzah

**Affiliations:** 1 Department of Computer Science and Technology, China University of Petroleum, Qingdao 266580, China; xuena_zhanghh@163.com; 2 Computer Vision Center, University Autònoma de Barcelona, 08193 Catalonia, Spain; poal@cvc.uab.es; 3 Laboratory MIA, University of La Rochelle, 17042 La Rochelle CEDEX, France; andrews.sobral@univ-lr.fr (A.S.); thierry.bouwmans@univ-lr.fr (T.B.); 4 Laboratory L3i, University of La Rochelle, 17042 La Rochelle CEDEX, France; ezahzah@univ-lr.fr; 5 School of Computer Science and Technology, Shandong University, Jinan 250100, China; chtu@sdu.edu.cn

**Keywords:** human pose estimation, human body models, generative methods, discriminative methods, top-down methods, bottom-up methods

## Abstract

Human pose estimation refers to the estimation of the location of body parts and how they are connected in an image. Human pose estimation from monocular images has wide applications (e.g., image indexing). Several surveys on human pose estimation can be found in the literature, but they focus on a certain category; for example, model-based approaches or human motion analysis, etc. As far as we know, an overall review of this problem domain has yet to be provided. Furthermore, recent advancements based on deep learning have brought novel algorithms for this problem. In this paper, a comprehensive survey of human pose estimation from monocular images is carried out including milestone works and recent advancements. Based on one standard pipeline for the solution of computer vision problems, this survey splits the problem into several modules: feature extraction and description, human body models, and modeling methods. Problem modeling methods are approached based on two means of categorization in this survey. One way to categorize includes top-down and bottom-up methods, and another way includes generative and discriminative methods. Considering the fact that one direct application of human pose estimation is to provide initialization for automatic video surveillance, there are additional sections for motion-related methods in all modules: motion features, motion models, and motion-based methods. Finally, the paper also collects 26 publicly available data sets for validation and provides error measurement methods that are frequently used.

## 1. Introduction

In Computer Vision, humans are typically considered as articulated objects consisting of rigidly moving parts connected to each other at certain articulation points. Under this assumption, human pose estimation from monocular images aims to recover the representative layout of body parts from image features. Extracted human poses are being used to analyze human behaviors in smart surveillance systems, to control avatar motion in realistic animations, to analyze gait pathology in medical practices, and to interact with computers, to cite but a few applications.

Traditionally, a human body pose can be accurately reconstructed from the motion captured with optical markers attached to body parts [1]. These marker-based systems usually use multiple cameras to capture motions simultaneously. However, they are not suitable for real-life non-invasive applications, and the equipment is quite expensive, confining their applications to lab experiments or long-term very costly productions such as controlling avatars’ movements in animations [2].

So, an increasing number of studies have been focused on markerless methods. The inputs are also captured by cameras, but the acting humans are not bound to wear any markers. Several types of images can be captured: RGB or grayscale images (which are the input image types we discuss in this survey), infrared images [3], depth images [4], and others. RGB images capture visible light, and are the most frequently seen images on the web; infrared images capture infrared light; and depth images contain information regarding the distance of objects in the image to the cameras. Infrared images are extremely useful for night vision, but are not in the scope of this review.

While ordinary cameras can capture RGB images, depth images require specialized equipment. This equipment is much less expensive compared with those for acquiring motion capture data, and can be used in everyday life settings. Commercial products include Microsoft Kinect [5], the Leap Motion [6], and GestureTek [7]. These products provide application programming interfaces (APIs) to acquire depth data [8]. The human pose detection problem has seen the most success when utilizing depth images in conjunction with color images: real-time estimation of 3D body joints and pixelwise body part labelling have been possible based on randomized decision forests [9]. Estimation accuracy from depth images are comparatively more accurate, but these devices can only acquire images within a certain distance limit (around eight meters), and a vast majority of pictures on the web are RGB or grayscale images with no depth information.

Human pose detection from a single image is a severely under-constrained problem, due to the intrinsic one-to-many mapping nature of this problem. One pose produces various pieces of image evidence when projecting from changing viewpoints. This problem has been extensively studied, but is still far from being completely solved. Effective solutions for this problem need to tackle illumination changes, shading problems, and viewpoint variations. Furthermore, human pose estimation problems have specific characteristics. First, the human body has high degrees of freedom, leading to a high-dimensional solution space; second, the complex structure and flexibility of human body parts causes partially occluded human poses which are extremely hard to recognize; third, depth loss resulting from 3D pose projections to 2D image planes makes the estimation of 3D poses extremely difficult.

In this paper, we collect milestone works and recent advancements in human pose estimation from monocular images. The papers in the reference section were downloaded during the first semester of 2016 from the following sources: Google Scholar, IEEE Explore, Scopus Elsevier, Springer, Web of Science, Research Gate, arXiv, and several research lab homepages. Each section of the paper is a possible component of human pose estimation algorithms. The flow of the sections follows the degree of abstraction: starting from images of low abstraction level to semantic human poses of high abstraction level.

Summarizing related works, there are two main ways to categorize human pose estimation methodologies [10]. The first way clusters solutions based on whether the human pose estimation problem is modeled as geometric projection of a 3D real-world scene or if it is treated as a general classification/regression problem. In geometric projection modeling (Section 4.1.2), a 3D human body model is required (Section 3.3). Furthermore, camera parameters are required for a projection model. From an image processing perspective, human pose estimation can be treated as a regression problem from image evidence.

In discriminative methods (Section 4.1.1), distinctive measurements, called features, are first extracted from images. These are usually salient points (like edges or corners) which are useful characteristics for the accomplishment of the estimation task. Later on, these salient points are described in a systematic way, very frequently statistically. This procedure is named “feature description”. In this review, we fuse feature extraction and feature description procedures into a feature section (Section 2). Instead, we categorize features based on their abstraction level: from low-level abstraction to high-level abstraction. Features of high abstraction levels are semantically closer to the human description of a human pose. Features are then assembled based on a predefined human body structure (Section 3.1 and Section 3.2) and then the assembled information is fed to a classification or a regression model to predict human body part layout. Then, various mapping models between extracted features and human poses are utilized (Section 4.1.1).

The second approach to categorization splits related works into top-down (Section 4.2.2) and bottom-up (Section 4.2.1) methods based on how pose estimation is carried out: if it introduces high-level semantics for low-level estimation or if human poses are recognized from pixel-level image evidence. There are also works taking advantage of different types of approaches simultaneously by fusing them to achieve a better estimation accuracy (Section 4.1.3 and Section 4.2.3).

One straightforward application of monocular human pose estimation is the initialization of smart video surveillance systems. In this scenario, motion cues provide valuable information, and progress in motion-based recognition could be applied to enhance pose estimation accuracy. The advantage is that an image sequence leads to the recognition of higher-level motions(like walking or running) which consist of a complex and coordinated series of events that cannot be understood by looking at only a few frames [11,12,13], and these pieces of higher-level information could be utilized to confine low-level human pose estimation. Extracted motion features are introduced in Section 2.4, human motion patterns extracted as motion priors are explained in the last paragraph of Section 3.4, and motion-based methods are described in Section 4.3.

**Figure 1:**
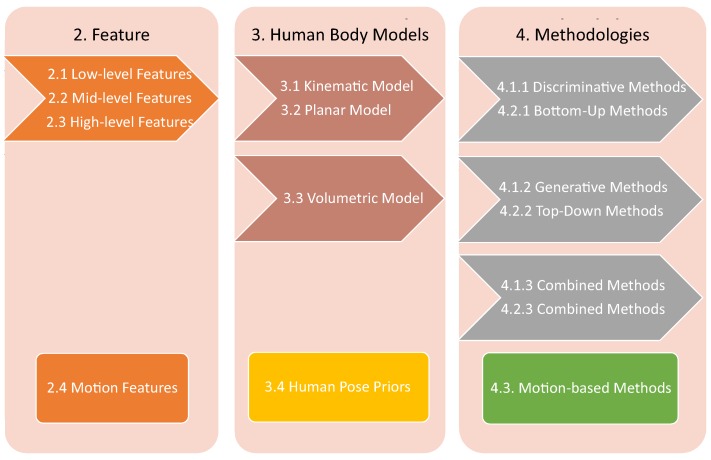
The Composition of The Review. The survey considers three processing units, and dedicates one section to each. After these three processing units, human poses can be estimated from images. Each directed flow chart denotes the composition of specific types of methods. Rectangle units are motion-based components.

The main components of the survey paper are illustrated in Figure 1. As mentioned before, it is not compulsory for a human pose estimation algorithm to contain all three components (features, human body models, and methodologies). For example, in Figure 1, the first flow line denotes three components of discriminative methods and bottom-up methods, including three feature types of different abstraction level, two types of human body models, and their methods. Temporal information provides motion-based components. In Section 5, we collect publicly-available datasets for the validation of human pose estimation algorithms, several error measurement methods, and a toolkit for non-expert users to use human pose estimation algorithms. Lastly, in Section 6, we discuss open challenges in this problem.

### 1.1. Related Works

Several surveys of human pose estimation can be found in literature. The authors of [14,15,16,17] give surveys of vision-based human pose estimation, but these works were conducted before 2009. A more recent comprehensive survey is from Liu et al. [18]. This survey studied human pose estimation from several types of input images under various types of camera settings (both single-view and multiple-view), and includes 104 references. In our survey, more than 300 references are included, and these works concentrate on a specific type of input: monocular images.

Other recent surveys were carried out on specific methodologies.For example, the survey from Lepetit et al. [19] and the survey from Perez-Sala et al. [20] both study model-based approaches, which employ human body knowledge such as the human body’s appearance and structure for the enhancement of human pose estimation. There are also surveys dedicated to human motion analysis where motion information is prerequisite [15,16,21,22].

An area that is closely related to human pose estimation is action recognition. Although algorithms and techniques used in human action recognition are different from those used in human pose estimation, recognition results of these two are sometimes combined within a framework to boost the performance of a single task [23,24,25,26]. Surveys on action recognition include [27,28,29,30].

### 1.2. Contributions

The past few decades have witnessed significant progress in human pose estimation, especially in recent years; deep learning has brought advancements in many research areas, including human pose estimation. The aim of this survey is to comprehensively overview milestone works on human pose estimation from monocular images for novices and experts in the field. Compared with past works, this review has the following contributions:

1. The first comprehensive survey of human pose estimation on monocular images including more than 300 references. These works includes top conferences and journals, which are milestone works on this topic. Table 1 gives a preview of included references, and its structure follows the composition of this paper. This survey considers several modules: features, human body models, and methodologies—as shown in Figure 1. We collect 26 publicly available data sets for the evaluation of human pose estimation algorithms. Furthermore, various evaluation measurements are included so that researchers can compare and choose an appropriate one for the evaluation of the proposed algorithm.
2. The first survey that includes recent advancements on human pose estimation based on deep learning algorithms. Although deep learning algorithms bring huge success to many computer vision problems, there are no human pose estimation reviews that discuss these works. In this survey, about 20 papers of this category are included. This is not a very large number compared to other problems, but this is a inclusive survey considering the relatively few works addressing this problem.

## 2. Features

Given monocular images, a very important question, and most frequently the first step in the pipeline, is to extract key points, describe them, and feed to the next processing unit. The performance of various features needs to be evaluated in order to determine which feature to choose within a certain context.

**Figure 2:**
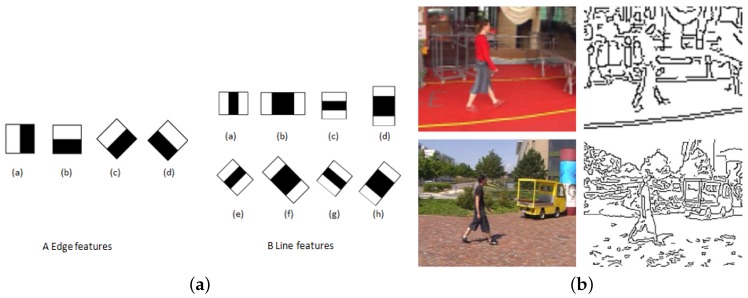
Edge Filter and Extracted Edge Feature Examples. (**a**) Haar Filters as Edge Filters; (**b**) Edge Features in [37].

Feature points extract most of the representative information in images, but are usually noisy and contain redundant information (as shown in Figure 2b). These features are then encoded to be more concise and descriptive. According to how the feature is encoded, the following sections are organized as follows: Section 2.1 presents low-level features which use extracted features directly; Section 2.2 describes preliminary feature encoding; and Section 2.3 introduces high-level features which denote semantic interpretation of image contents. In low-level features, both features measured in the vicinity of described points and features describing overall characteristics of a target are considered.

### 2.1. Low-Level Features

To capture appearance, geometry, and shape information of human body parts, features commonly extracted are silhouettes [31,32,33,34], contours [35,36], edges [37,38], etc. Silhouettes extract outlines of objects and are invariant to texture and lighting [32,128,224,225,226]. Contour captures the outline of body parts and is a path with edges linking crossing points of segmentation boundaries [36]. Edges extract sharply varying lines in images and are usually computed by convolution.

**Figure 3:**
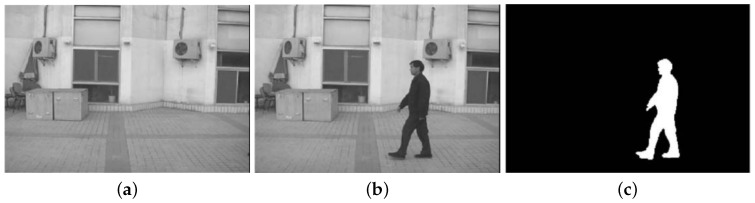
Examples of Silhouette Extraction in [227]. (**a**) The background image; (**b**) An original image; (**c**) The extracted silhouette from (**b**).

**Figure 4:**
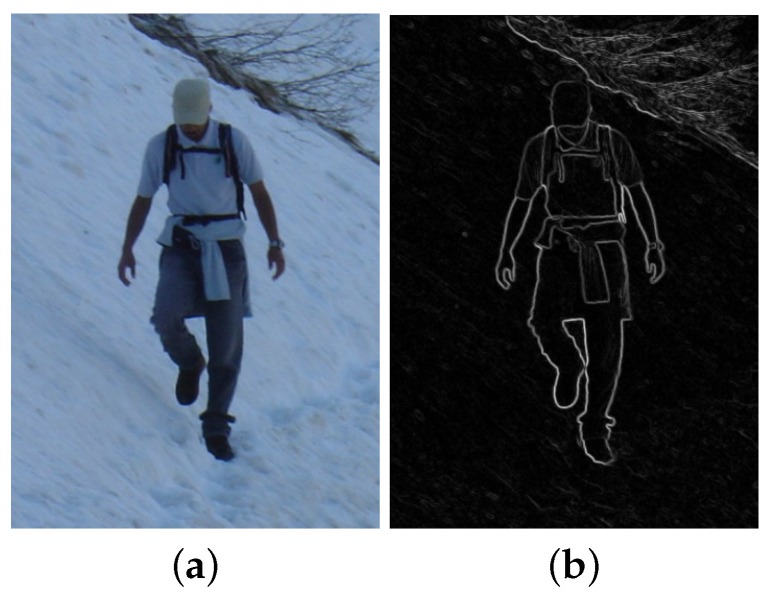
Contour Features from [228]. (**a**) An original image; (**b**) Extracted contours.

In comparison, silhouettes are global descriptors enclosing an overall view of an object and usually require prior knowledge of the background to extract the foreground object, as shown in Figure 3; Contours require pre-processing (such as segmentation), and they enclose details in addition to outline information, as shown in Figure 4; Edges are rather scattered features and can be computed directly from filtering, as shown in Figure 2b. Figure 2b shows examples of edge filters for convolution and detected edge examples in [37]. Figure 2a shows Haar features as an example of edge and line filters. Other features that model body part appearance include color [36,39,40] and texture [41].

### 2.2. Mid-Level Features

**Figure 5:**
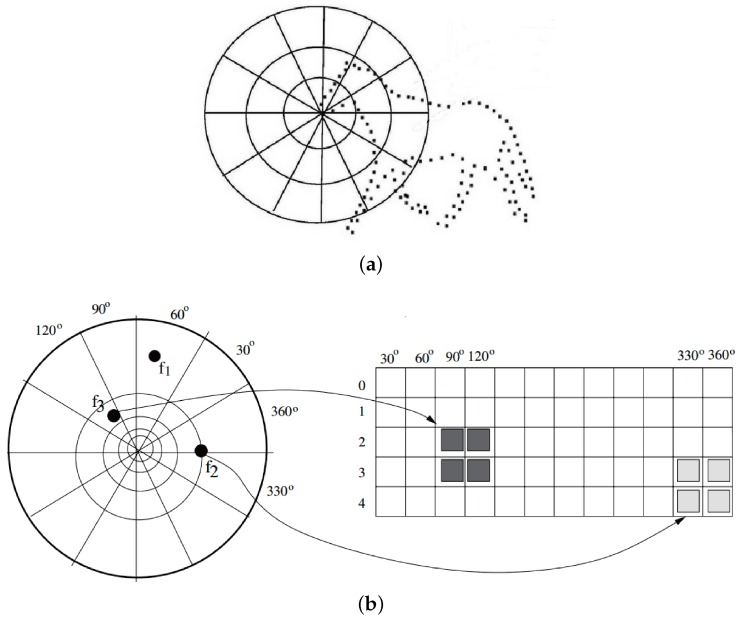
Shape context examples. (**a**) Log-polar coordinates in shape context; (**b**) Shape Context Encoding.

Extracted silhouette features are usually encoded as Fourier descriptors [42], shape contexts [44], geometric signatures [48], Poisson features [49], and so on. The most frequently used shape context descriptor captures the distribution of points relative to the current point being described, as shown in Figure 5a. Specifically, a histogram is computed using log-polar coordinates, and the space is divided into several angle and radius bins. Points falling in each bin are accumulated to form a histogram distribution, as shown in Figure 5b. It converts distributed points into a multi-dimensional descriptor, and this statistical means of computation is robust against local silhouette segmentation errors [43,44,45,46,47].

**Figure 6:**
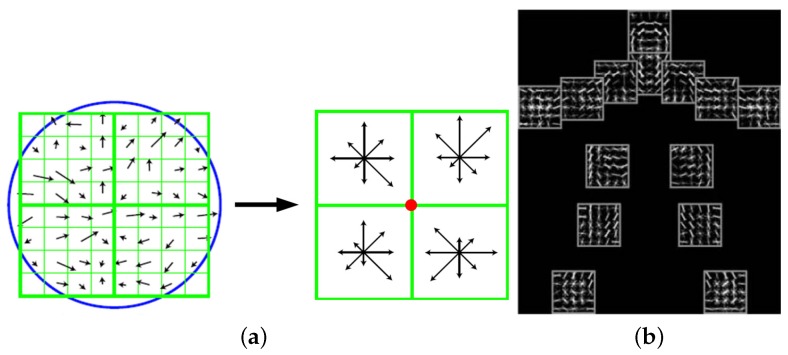
Two widely utilized feature extractors and descriptors. (**a**) Scale Invariant Feature Transform (SIFT); (**b**) Histogram of Gradient (HOG) templates [229].

**Figure 7:**
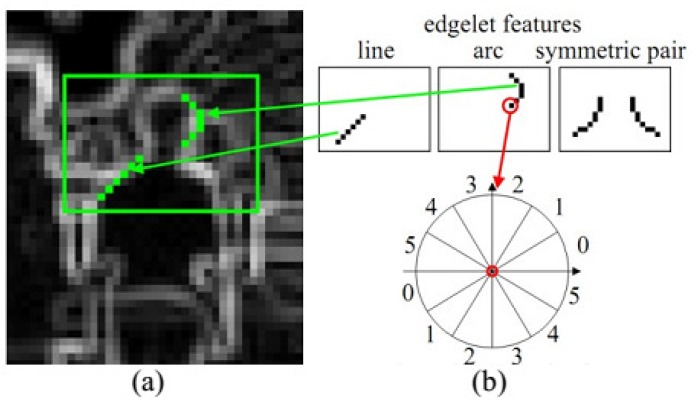
Edgelet features [230]. (**a**) The Sobel convolution result; (**b**) Examples of edgelet features and orientation quantization.

**Figure 8:**
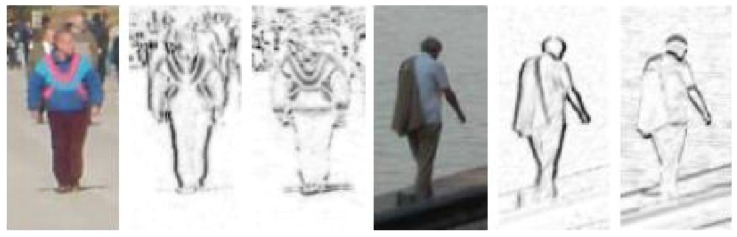
Shapelet Features from Two Sample Images. Each computed in one direction [59].

Other features based on edges or gradients are encoded as histograms of oriented gradients (HOG) [50,51,52], relational edge distribution [53], Scale Invariant Feature Transform (SIFT) [54,55] and SIFT-like features [56,57], edgelet features [58], shapelet features [59], and so on. By measuring on a number of scales, SIFT features (shown in Figure 6a) can be matched against scale variance and are extremely popular among computer vision researchers before deep convolution networks are widely applied to automatically extract features. HOG features are extremely popular features for human pose estimation, and usually several HOG templates representing various states of a body part are learned (visualized in Figure 6b). Edgelet (in Figure 7) and shapelet (in Figure 8) features are combinations of edges and gradients, respectively.

Other than local features mentioned above, there are many global features which capture overall characteristics, for example, the object foreground map [46] and dense grid features, like the grids of HOG descriptors [50] or grids of SIFT features [56,60]. Grid features—for example, grid of SIFT—outperform the SIFT feature extractor and descriptor, according to experience.

Multilevel hierarchical encodings, like Hierarchical Model and X (HMAX) [61], hyperfeatures [62], spatial pyramid [63], vocabulary tree, and Multilevel Spatial Blocks (MSB) [64] are more stable in preserving invariance to geometric transformations. Other features, such as local paths [231], prediction pipeline [232], and Extremal Human Curves [233] are also common features in human pose estimation.

A convolutional neural network (CNN, or ConvNet) is currently the most popular feature in computer vision, artificial intelligence, machine learning, and many other fields. CNN is an extension of a neural network. Input images are processed by convolution and downsampled several times to extract features, and fully-connected layers consider integrated efforts from all. Estimated errors are back-propagated, and network parameters are adjusted accordingly. Recently, many works have used CNN extracted features for human pose estimation [65,66,67].

### 2.3. High-Level Features

Several descriptors have high-level characteristics, such as body part patches, geometry descriptors, or context features. Body part patches assume any of the spaced orientation, and they can have any position inside the patch. They are more general descriptors compared to body parts, which are confined within a body limb, between body joints, or within the vicinity of a body joint. The combined body parts, as a geometry descriptor, contain semantic relations among single parts [68,69,70], usually encoded as putting two sets of features together, including body parts’ location and orientation [36]. Context, on the other hand, captures spatial or temporal correlations, and can represent task-specific features [8]. High-level features encode semantic co-occurrence between composing units. Compared with mid-level features, which are a spatial or temporal encoding in a predefined pattern, high-level features mine correlations from training data and let data speak for itself.

### 2.4. Motion Features

As mentioned previously, estimated poses from monocular images could be utilized as an initialization for pose tracking in smart surveillance systems. Temporal and spatial consistency in videos could be extremely useful; for example, it can be used to correct estimation failure in one single frame. We review motion cues utilized by human pose estimation.

**Figure 9:**
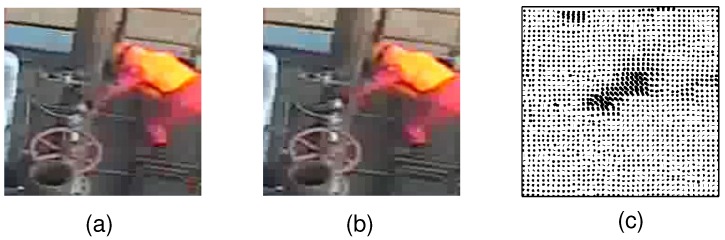
Illustration of the Optical Flow Descriptor. (**a**,**b**) Reference images at time *t* and *t* + 1; (**c**) Computed optical flow.

Motion features such as dense optical flow [71], robust optical flow [72], edge energy and motion boundaries, and their combinations [73] enhance estimation performance by temporal correspondence. Optical flow [234] is the pattern of object, surface, and edge motions caused by the relative motion between an observer and the scene (shown in Figure 9). The gradient in the optical flow is related to movements, and could be used to track poses [213,235]. Features representing local motion similarities, such as motionlet [151,152] and motion and appearance patches based on image difference [236] are also used.

Single features are insensitive to background variations, thus resulting in ambiguities. Features can be combined to improve the performance of pose estimation [237,238]. Human poses in monocular images could be estimated more accurately by combining multiple image cues with different traits, such as edge cue, ridge cue, and motion cue [239].

## 3. Human Body Models

One of the key issues in human pose estimation is how to build and describe human body models. A human body encloses human body kinematic structure information, human body shape information, and texture information, if possible. For example, a kinematic joint model of around 30 joint parameters and eight internal proportion parameters encoding the positions of the hip, clavicle, and skull tip joints, and the human body shape can be denoted as nine deformable shape parameters for each body part, gathered into a vector [226]. In discriminative methods, the kinematic models are utilized to assemble separately detected body parts or body joints. Under geometric projections, these models with a pose can be mapped to a plane, and thus compare with image evidence to verify the projected pose.

**Figure 10:**
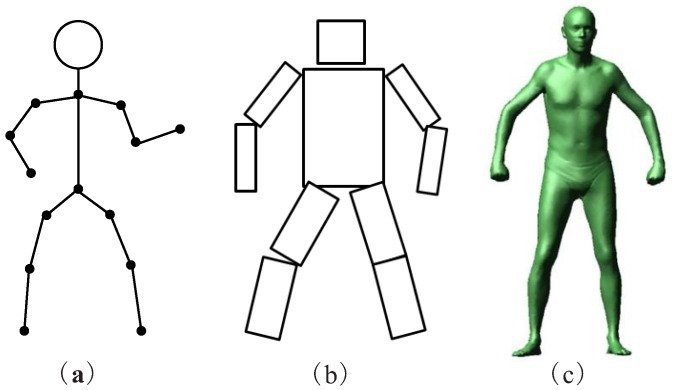
Three Types of Human Body Models. (**a**) Kinematic model; (**b**) Cardboard model; (**c**) Volumetric model.

The configuration of a human pose can be determined by body part orientation. A stick is capable of specifying a limb orientation, thus a human body can be modeled as a stick figure—as shown in Figure 10a. Body part volumes play an important role in localization when the volumetric human model (as shown in Figure 10c) needs to be projected onto a 2D image plane where the effectiveness of the pose is validated by comparing with image evidence. In the following sections, we discuss various types of human body models.

### 3.1. Kinematic Model

Models that follow the skeletal structure are called kinematic chain models [91]. The set of joint positions and limb orientations are both effective representations of a human pose. One coordinate-free representation is introduced in [137]: the local coordinates of the upper-arms, upper-legs, and the head can be converted into spherical coordinates, and the discretized azimuthal and polar angles of the bones can be defined. The kinematic model allows us to incorporate prior beliefs about joint angles. To achieve this, a set of joint angle training data needs to be labelled with positive and negative examples of human pose [108].

There are two categories of the kinematic model; one is the predefined model, and the other is the learned graph structure. A very popular graph model is pictorial structure models (PSM) [71,74]. A special case of PSM is tree-structured models. Thanks to their unique solutions, tree-structured models are successfully applied in human pose estimation, in either 2D or 3D [41,75,76,77,78,79,80,81]. However, the inference is unable to capture additional dependencies between body parts, other than kinematic constraints between connected parts. For example, a kinematic tree model has its limitations in representing global balance and gravity constraints. In addition, the body parts could not be completely detected under the circumstance of partial occlusion [240].

Many researchers seek an improvement of tree-structured models [36,82,83,84,85,86,87,88,89]. For instance, authors in [82] solve the lack of model description by adding tree-structured models with different shapes, the authors of [83] add the spatial constraint of unconnected body parts by changing the optimized objective function, the authors of [88] enhance the descriptive ability by adding the states of the models. The authors of [82] use multiple tree models instead of a single tree model for human pose estimation. The parameters of each individual tree model are trained via standard learning algorithms in a single tree-structured model. Another example of using multiple tree structures is [241], where different tree models are combined.

More general than predefined structure models, pairwise body part relations could be learned from images [90]. Additionally, a tree structure based on Bayesian networks could be learned [91,92]. These models are non-parametric with respect to the estimation of both their graph structure and their local distributions.

### 3.2. Planar Model

Other than capturing the connecting relations between body parts, planar models are also capable of learning appearance. Various means are used to learn the shape and appearance of human body parts. One example is Active Shape Models (ASMs). ASMs are used to represent the full human body and capture the statistics of contour deformations from a mean shape using principal component analysis (PCA) [93,94,95,96].

Another example is the cardboard model (shown in Figure 10b), composed of information about object foreground colors and body part rectangular shapes. The cardboard model usually has a torso and eight half limbs, each body part’s appearance is represented by the average RGB color, and the foreground color histogram is also stored. For example, the authors of [97] used the cardboard model for human pose estimation.

### 3.3. Volumetric Model

Volumetric Models realistically represent 3D body shapes and poses. Geometric shapes and meshes are both effective volumetric models. When using geometric shapes as model components, human body parts are approximated with cylinders, conics, and other shapes, assembling body limbs. For example, a person could be modeled as a composite of cylinders, with each cylinder connected to one or several other cylinders [98]. Each joint of the cylinders has 1 to 3 degrees of freedom (DOF). The model is described by the global translation and rotation. The limb pattern is extracted from the model parameters, and the surface space can be determined by solving the least-square problem [242]. Conic sections are also utilized to model 3D human limb shapes. Cylindrical and conic sections lead to rectangular or quadrilateral projected shapes. Such models clearly capture the true shape of human limbs given wide variations in anatomy or clothing, and are more accurate than pictorial structure-based approaches.

Another way of modeling a volumetric human body is meshes. The meshes are deformable and triangulated models, so they are more suited for the representation of non-rigid human bodies [106]. One way to acquire mesh models is through 3D scans [243,244,245]. To estimate joint locations, the meshes are usually segmented to several body parts. One widely-used 3D mesh model is Shape Completion and Animation of People (SCAPE) [99,100,101,102,103]. Stitched puppet [104] models enhance the SCAPE model by adding pairwise potentials. They define a “stitching cost” for pulling the limbs apart, and learn pairwise relationships from images.

Furthermore, 3D human body models are incorporated with shading. For a given mesh, the shape deformation gradients are concatenated into a single column vector. A Blinn–Phong model with diffuse and specular components can be used to approximate a body’s reflectance when there is a single light source [246]. The shadows cast from a point light source provide additional constraints on pose and shape [105]. After the pose and shape parameters are estimated, the light position from shadows are determined, and the pose and shape from foreground regionsand shadow regions are also re-estimated.

Models that are expressive enough to represent a wide range of human bodies and poses with low dimensions are also explored [94]. The authors of [99] build on the SCAPE model and develop a factored representation.

### 3.4. Human Pose Priors

The human body pose is constrained by several factors, such as kinematics, operational limits of joints, and behavioral patterns of motion in specific activities [247,248]. Kinematic constraints, together with a dynamic model, provide enough information to estimate human poses [249].

The availability of motion capture techniques [250,251,252] allows pose priors to be learned from data. To learn pose constraints efficiently, the authors of [107] collect a motion capture data set to explore human pose possibilities. With collected data, a set of joint angle training data labeled with positive and negative examples of human poses could be utilized [108]. However, pose priors learned from one motion have problems generalizing to novel motions [110].

Some studies learn the human pose priors as a pose-dependent model of joint limits [111], and others train random forests (RFs) and principal direction analysis to model the human bodies [2]. For physics-based models with dynamics, related works include [112,113]. When temporal information is available, prior models [109] of human motion can be learned to constrain the inference of 3D pose sequences to improve monocular human pose tracking.

## 4. Methodologies

There are two main ways of categorizing human pose estimation algorithms. Based on whether human pose estimation is modeled as a geometric projection or is treated as a specific image processing problem, related works can be classified into two main groups: generative methods or discriminative methods.

Another way of categorization differentiates between whether the human pose estimation problem is worked out by beginning with a high-level abstraction and working downwards or by beginning with low-level pixel evidence and working upwards. Methods working downwards are called top-down methods, while bottom-up methods work upwards.

### 4.1. Discriminative Methods and Generative Methods

**Figure 11:**
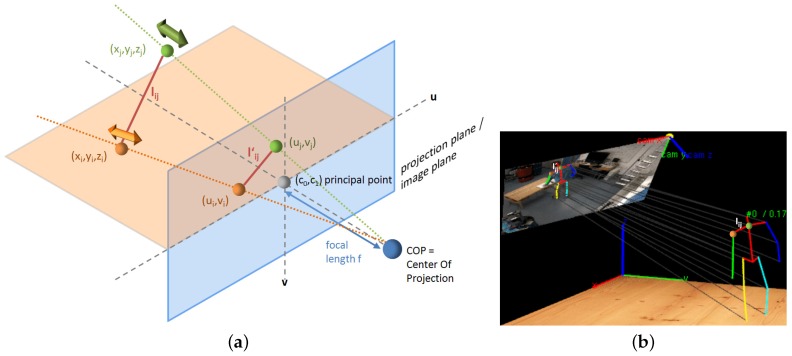
Geometric Reconstruction of 3D Poses. (**a**) Perspective camera models; (**b**) An example pose and its projection.

The generative model is defined in terms of a computer graphics rendering of poses. A volumetric human body model is usually required, and the model is projected to image space (as shown in Figure 11a) and adjusted so that the projection and the image observation are compliant (as shown in Figure 11b). While in learning methods, correspondences between image features and human poses are modeled, and the 3D human pose estimation problem is treated as a search or a regression problem. The learning method is usually faster, as it considers only image observations, while the generative method models the intrinsic process of this problem. The discriminative model consists of a set of mapping functions that are constructed automatically from a labeled training set of body poses and their respective image features.

One of the differences between generative methods and discriminative methods is that the first category starts from a human body model initialized with a pose and projects the pose to the image plane to verify with image evidence (as shown in Figure 11b), while the second category starts from the image evidence and usually learns a mechanism modeling the relations between image evidence and human poses based on training data. Their working directions are completely opposite.

#### 4.1.1. Discriminative Methods

Discriminative approaches start from the image evidence, estimate pose by a mapping- or a search-based mechanism. The model describing the relations between the image evidence and the human poses could be learned from training data [253]. Once the model is trained, testing is usually faster than generative methods, because it descends into a formulation calculation or a constrained search problem instead of optimizing a high-dimensional parametric space. Discriminative approaches search for the optimal solutions within their scope [254,255,256,257,258,259].

**Figure 12:**
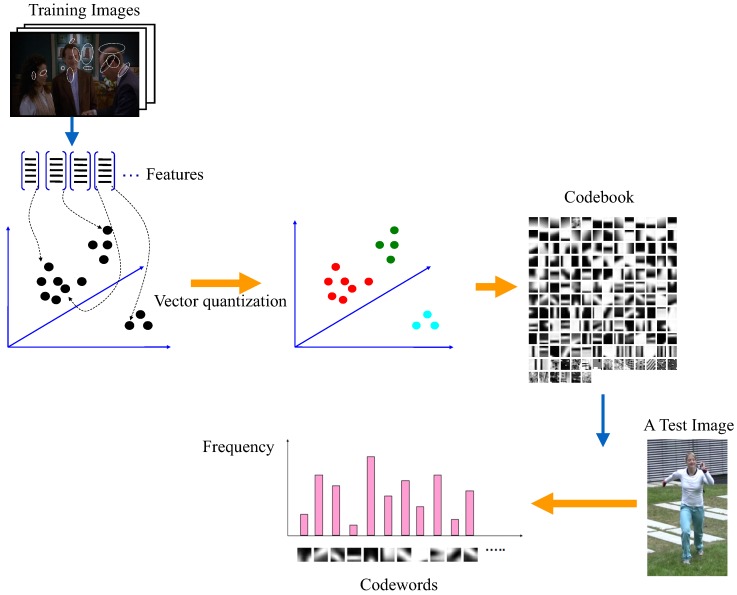
Bag-of-words feature representation pipeline.

**Figure 13:**
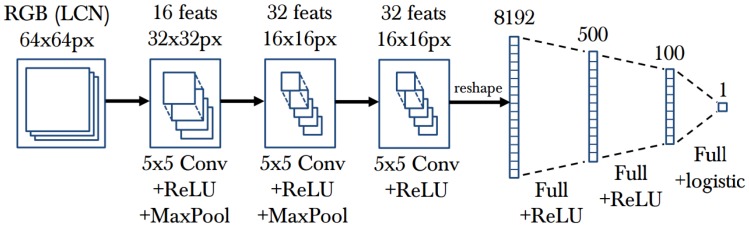
The convolutional network architecture used in [156]. It includes: one input layer, two convolution and down sampling layers, one convolution layer, two fully connected layers, one logistic regression layer, and one output layer. Note, “LCN” stands for local contrast normalization, and ReLU and logistic are activation functions.

**Figure 14:**
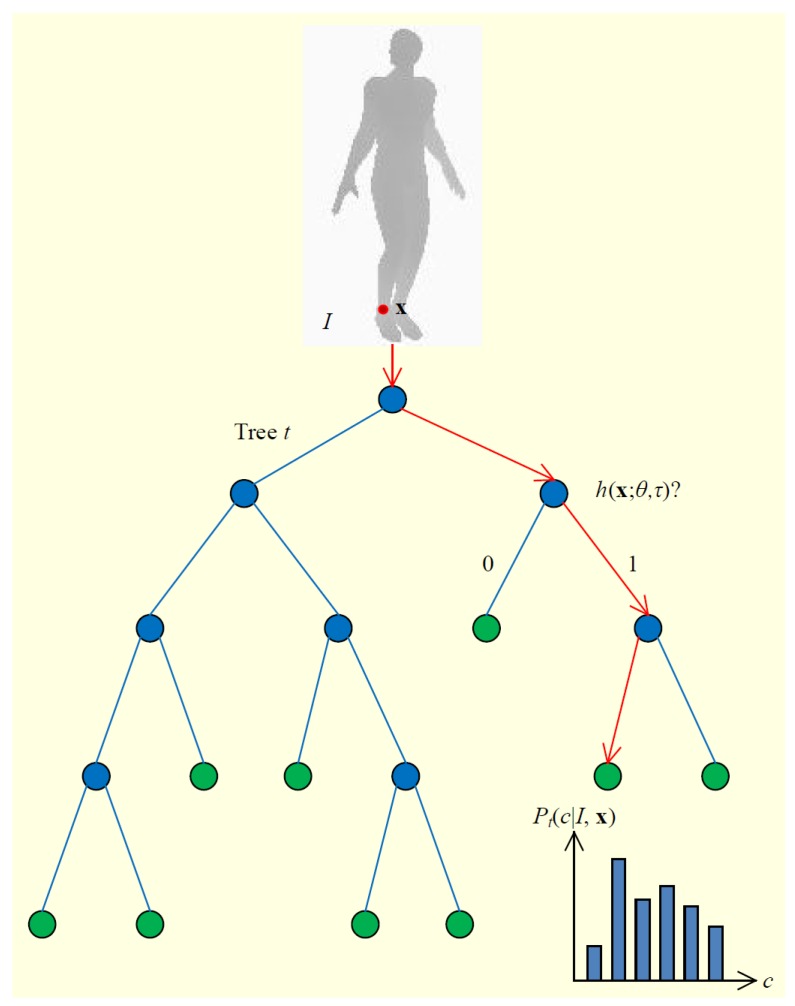
A tree that composes random forests [167]. The tree consists of split nodes (blue) and leaf nodes (green). The red arrows indicate the path that is taken for a particular input.

There have been many studies utilizing this category of methods, and they can be further divided into two main sub-categories: learning-based [34,160] and example-based [260,261] methods. These sub-categories are further divided as follows:

1. **Learning-based methods**
  (a) Mapping based methods. One extremely popular model for learning these types of maps isSupport Vector Machine. Support Vector Machines (SVMs) [120,121,122] are discriminant classifiers that train hyperplanes for discrimination between classes. The most decisive examples in training are picked as support vectors. Similarly, in Relevance Vector Machines (RVMs), which are a Bayesian kernel method, the most decisive training examples are picked as relevance vectors [32,123,124,125]. Non-linear mapping models are also utilized, for example, Gaussian Processes [26].
    More complex mapping mechanisms can be modeled with a Mixture of Experts (MoE) model, a Bayesian mixtures of experts (BME) model, and other models. For example, the authors of [262] exploit a learned MoE model which represents the conditionals [126,127,128] to infer a distribution of 3D poses conditioned on 2D poses. BME [129,130] could model the multi-model distribution of the 3D human pose space conditioned on the feature space, since the image-to-pose relation is hardly linear.
    Mapping-based methods can also be further categorized into direct mapping methods and 2D-to-3D boosting methods. One class of learning approaches uses direct mapping from image features [32,60,131,132,133,162,263], and another class of approaches maps the image features to 2D parts and then uses modeling or learning approaches to map 2D parts to 3D poses [78,134,135,136,137].
    Based on whether the mapping is learned with labelled ground truth data or not, mapping can be both supervised and unsupervised [64,138]. Furthermore, semi-supervised methods are used as well [139,140,141].
  (b) Space learning-based methods. Both topology space and subspace are utilized to learn mapping. For example, in a topology space-based method, arbitrary non-rigid deformations of a 3D mesh surface could be learned as manifold [24,34,142,143,144,145,146,147].
    On the other hand, subspace could also be learned to constrain the solution space. For example, an embedding can be learned by placing images in similar poses nearby, avoiding the estimation of body joint positions [148,149]. Dimensional reduction technologies can also be used to remove redundant information [150]. Locality-constrained Linear Coding (LLC) algorithms [151,152] can also be performed to learn the nonlinear mapping in order to reconstruct 3D human poses.
    Other methods, such as Relevant Component Analysis (RCA) [64], Canonical Correlation Analysis (CCA), and Non-negative matrix factorization (NMF) [56] are also typical algorithms used to mine data correlations.
  (c) Bag-of-words based methods. The bag-of-words pipeline is the most popular computer vision algorithm solution before the deep learning algorithm. The main idea of the bag-of-words pipeline is to first extract the most representative features as a vocabulary, and then denote each training data based on image evidence and the vocabulary in a statistical way: the occurrence of each word in the image is counted, all occurrences of words in the vocabulary form a histogram, and this histogram is taken as the final representation of the input image. This representation process is shown in Figure 12. This feature representation is then fed to a classifier or a regression model to complete the task [130].
    By selecting the most representative features as the vocabulary, followed by a histogram representation based on the vocabulary, an image can be represented with a vector of a fixed length equal to the size of the vocabulary. In this way, the image is represented with a statistical occurrence of the most salient features and is compressed to the size of the vocabulary.
  (d) Deep learning-based methods. Deep learning is an end-to-end learning method that automatically learns the key information in images. Convolutional Neural Networks (CNN) [156,157,264,265] are popular deep learning models which have multi-layers, with each layer composed of multiple convolutions and some other hybrid architectures (refer to Figure 13 for an example of CNN architecture). Deep learning-based human pose estimation mainly has three categories: (1) combined part detection with the accurate localization of human body parts through deep learning networks [66,153,154]; (2) learning features through deep convolutional neural networks and learning human body kinematics through graphical modelling [155,156]; (3) learning both features and body part locations through deep learning networks [90,157,158,159].
    The regression methods [162] based on deep learning have various extensions, such as a mixture of Neural Networks (NNs) [160] which uses a two-layer feedforward network and linear output neurons as a model for local NN regression. The authors of [155] also propose a combined architecture that involves a deep convolutional network and a Markov Random Field (MRF) model. The authors of [163] present a CNN that involves training an Regions with CNN features (R-CNN) detector with loss functions. The authors of [164] adopt an iterative error feedback that changes an initial solution by feeding back error predictions.
2. **Exemplar-Based Methods**
  The exemplar-based approaches estimate the pose of an unknown visual input image [118] based on a discrete set of specific poses with their corresponding representations [160]. Randomized trees [165] and random forests [166,167] are fast and robust classification techniques that can handle this type of problem [266].
  Random Forest is an ensemble classifier that consists of several randomized decision trees [142,267] and has a nonterminal node containing a decision function to predict the correspondences by regressing from images to terminal nodes, like mesh vertices [9] (Figure 14 shows an example). Enhanced random forests were used by [268], which employed two-layered random forests as joint regressors, with the first layer acting as a discriminative body part classifier and the second one predicting joint locations according to the results of the first layer.
  Another type of approach is based on Hough forests. Hough forests are combinations of decision forests, and the leaf nodes in each tree are either a classification node or a regression node. The set of leaf nodes can be regarded as a discriminative codebook. The authors iof [269] directly regressed an offset to several joint locations at each pixel. Improved versions include an optimized objective, like a parts objective (“PARTS”) based on discrete information gain [9], while other works report the generalization problem of the specified objective [270,271]. Furthermore, sparse representation (SR) is used to extract the most significant training samples, and later on, all estimations are carried out based on these samples [168,169,170,171].

#### 4.1.2. Generative Methods

The predictions made at the pixel level yield a set of independent local pose cues that are unlikely to respect kinematic constraints. By fitting a generative model to these cues, [142,272,273] resolve this problem.

Generative approaches [22,114,115,116,117,118,119] model the likelihood of the observations given a pose estimate. Inference involves a complex search over the state space to locate the peaks of the likelihood [128]. Generative methods are susceptible to local minima, and thus require good initial pose estimates, regardless of the optimization scheme used. The pose is typically inferred using local optimization [274,275,276,277,278] or stochastic search [279,280,281].

#### 4.1.3. Combined Methods of Discriminative and Generative Methods

Generative methods project the human model into the 2D image space and measure a distance between them [160], while the discriminative methods detect the parts of the human body to reconstruct the human pose. Generative methods suffer from low efficiency, while discriminative methods struggle to generalize to poses not present in the training data [130].

**Figure 15:**
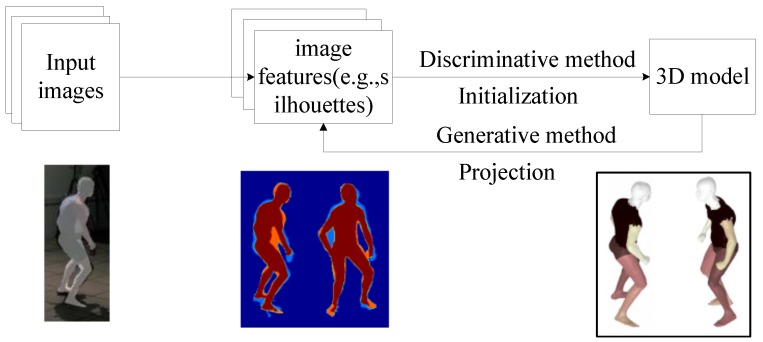
Overview of the combined method of discriminative and generative methods.

To take advantage of both categories and avoid their shortcomings, some research was done exploring the combination of these two types of methods together. The combination is generally implemented by initializing the pose with the estimation from discriminative methods [179] and optimizing the human pose within a local area through generative methods [172,173,174], as shown in Figure 15. Through iterative optimization in the generative process, poses of the 3D human model are adjusted by comparing with image evidence in the discriminative process.

In generative methods, the space of silhouettes can be projected from 3D human poses. One pose generates several different silhouettes under various viewpoints [175]. The structural parameters of the 3D articulated volumetric model contribute to the projection of the 3D geometric human body model [226,282], and Bayes’ rule could be used to estimate the model parameters and achieve a probabilistic interpretation. An estimated pose with the discriminative method could be used as initialization, and the manifold of silhouette space could be used to optimize the optimization [147,176].

Other combined methods include probabilistic Gaussian modelling and others [177,178,179]. These two models could also be combined to inference the articulated human pose by deriving a combined formulation [180].

### 4.2. Bottom-Up Methods and Top-Down Methods

We consider a second way to categorize, based on the direction human pose estimation algorithms are working semantically; that is, the method works from top level semantic abstraction to low level, or it works the other way around. Images are considered as the lowest level in the semantic hierarchy, human pose configuration is considered as in the higher level, and also human action types to which human poses belong. Note that some notations use top-down methods to refer to generative methods described above and use bottom-up methods to refer to discriminative methods. In this paper, we do not use these terms in this way.

#### 4.2.1. Bottom-Up Methods

**Figure 16:**
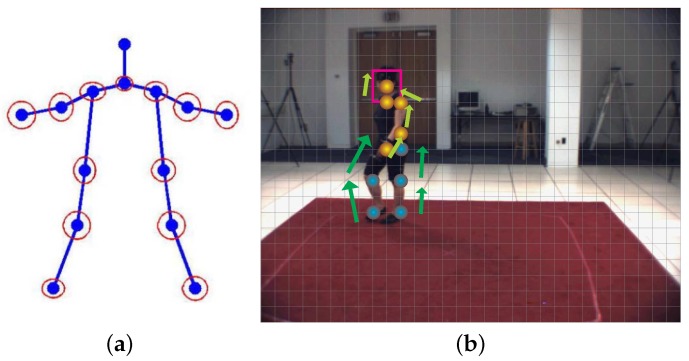
Tree-structured human body model in human pose estimation. (**a**) Tree-structured body model; (**b**) A pose estimation example.

In bottom-up methods, pieces of image evidence are collected and described to form descriptive features. These features are sometimes utilized directly to predict human poses, and sometimes used to localize body parts whose occurrences in images are then assembled to form a human occurrence. In Section 4.1, we discuss mechanisms modeling image representations and human pose correspondences. In this section, we collect and compare methods fusing low-level image evidence to form high-level semantics. Based on unit size, bottom-up methods can be further divided as follows:

1. **Pixel- or superpixel-based methods.** Pixel information can also be used to boost pose estimation accuracy [186]. For example, pixel information is used as input to an iterative parsing process, which learns better features tuned to a particular image [182].
  The pixels or superpixels of an image can also be used to formulate a segmentation function and be integrated into pose estimation. For example, they can be used to formulate the energy function of segmentation algorithms and integrate object segmentation with a joint optimization [187,191,193].
  Pixel-based methods can also be combined with other methods. For example, the authors of [192] extend the per-pixel classification method with graph-cut optimization, which is an energy minimization framework. Furthermore, results from segmentation can be utilized to enhance pixel-level estimation. The authors of [188] propose an approach that progressively reduces the search space for body parts by employing “grabcut” initialized on detected regions to further prune the search space [189,190]. Part-based and pixel-based approaches can also be combined in a single optimization framework [208].
  The superpixels are also useful in restricting the joint positions in the human body model [283]. In superpixel-based methods, body part matching and foreground estimation obtained by superpixel labeling could be optimized, for example, with a branch-and-bound (BB) algorithm [97,183,184,185]. Additionally, the authors of [284] compare the quality of segmentation derived from appearance models generated by several approaches.
2. **Part-based methods.** Part-based methods solve pose estimation problems through learning body part appearance and position models. In part-based methods, body part candidates are first detected from image evidence, and then detected body parts are assembled to fit image observations and a body plan [206]. As an iconic work, a flexible mixture of parts model was introduced in [80], which extends the deformable parts model (DPM) [41] for articulated 2D body pose estimation. It was further improved using a compositional and/or graph grammar model [285].
  One key issue in part-based methods is to decide how to fuse responses of each single body part into a whole, and this is related to how the human body is modeled. We organize the following based on the characteristics of the human body models, and further divide part-based methods.
  (a) Pictorial Structures. Pictorial structures [36,77,79,189,194,196,197,286] are a kind of graphical kinematic model over detection methods, with the nodes of the graph representing object parts, and edges between parts encoding pairwise geometric relationships.
    Different deformations of the classic Pictorial Structures models have been developed, such as Adaptive Pictorial Structures (APS) [79], Multi-person Pictorial Structures (MPS) [195], Poselet Conditioned Pictorial Structures [198], the Fields of Parts (FOP) [199], and others.
    The tree structure is one of the most successfully applied pictorial structures. The model decomposes a tree structure into unary appearance terms and pairwise potentials between pairs of physically-connected parts, as shown in Figure 16a. With sliding windows methods, trained body part templates (HOG templates are visualized in Figure 6b) are compared with image features. Responses from all body parts are passed through the tree structure (as shown in Figure 16b), and a final score is calculated at the root of the tree.
  (b) Enhanced Kinematic Models. Enhanced kinematic models often have better appearance, and are more expressive in describing pose constraints. For example, a variety of modes are included to enhance the representation abilities of the kinematic model, such as the Multimodal decomposable model (MODEC) model [88], which has a left and right mode and half- and full-bodied modes.
    There have also been many studies conducted on improving kinematic models with cascaded structures. For example, the authors of [36] propose a coarse-to-fine cascade of pictorial structure models. The states of cascade framework could be pruned and computed [201]. By resorting to multiple trees, the framework estimates parameters for all models, requiring only a linear increase in computation over learning or inference than a single tractable sub-model [200]. The authors of [84] propose a new hierarchical spatial model that can capture an exponential number of poses with a compact mixture representation on each part. Using latent nodes, it represents a high-order spatial relationship among parts with exact inference.
    Furthermore, instead of pre-defining a kinematic model, a latent tree model [287] can recover a tree-structured graphical model which best approximates the distributions of a set of observations. In addition, by modifying regression methods, pose estimation accuracy can be improved. For example, the authors of [187] introduce part-dependent body joint regressors to classify the body parts and predict joint locations.
    The local scores of children in tree-structured models could be correctly traversed to their parents, while in case occlusion, the score may traverse to the wrong parent, resulting in missing parts and inaccurate detection, turning the tree structure into a graph [288]. Enhanced tree-structured models are also proposed to deal with this problem. The occlusion rectification method based on regression could detect occlusion by encoding the kinematic configurations in a tree. Since non-adjacent parts are independent, the occluded parts could be estimated [289]. The problems of foreshortening and part scale variation can be addressed by defining a body part with body joints instead of body limbs [206,258,290].
    None-tree methods have recently been proposed to facilitate stronger structure constraints, and can be optimized using convex programming or belief propagation [130]. It is believed that loopy graphical models are necessary when combined parts are used to handle large variance in appearance [87]. Loopy Graphical Models [202,203] begin by sending messages from the leaf nodes to the root, and then from the root node to the rest. Articulated grammar models are another example of non-tree models. The authors of [285] present a framework using the articulated grammar model to integrate a background model into the grammar to improve localization performance.

#### 4.2.2. Top-Down Methods

The top-down method is used to refer to generative methods in [181,291], but in this survey we use this term to denote the problem solving process of working from high-level semantic to lower-level image evidence [181], where high-level semantic is used to guide low-level recognition. By this notion, top-down methods are more frequently combined with bottom-up methods than being used as a separate method, since higher-level semantics are usually what we want to achieve.

#### 4.2.3. Combined Bottom-Up and Top-Down Methods

The way that bottom-up methods and top-down methods combine is more flexible than the way discriminative and generative methods combine:

1. **Combined detection- and recognition-based methods.** Motivated by extensive literature on both detection [33,35,51,58,59,200] and recognition [32,52,236,260,292,293,294], many works explore the possibility of combing these two types of methods together to enhance estimation accuracy [37,204]. For example, by combining the graphical kinematic models with detection methods, the detection and 3D poses could be obtained simultaneously [60,205,206,207]. On the other hand, the authors of [295] introduce a method of monocular 3D pose estimation from video using action detection on top of a 2D deformable part.
2. **Combined pixel-based and part-based methods.** Concurrent optimizing object matching and segmentation enables more robust results, since the two closely-related pixel-based and part-based methods support each other [46,193,208]. For example, pixel-wise body-part labels can be obtained by combining part-based and pixel-based approaches in a single optimization framework [208].
  The authors of Bray et al. [205] use graph cuts to optimize pose parameters to perform integrated segmentation and 3D pose estimation of a human body. Global minima of energies can be found by graph cut [209], and the graph cut computation is made significantly faster by using the dynamic graph cut algorithm [210].

### 4.3. Motion-Based Methods

With temporal information, human pose estimation could be boosted with temporal and spatial coherence, and human pose estimation could also be considered as human pose tracking. In this case, not only body part shape and appearance are learned, but body part motion should also be extracted. With motion cues, the articulation points of the human body can be estimated by the motion of the rigid parts, and the constraints between adjoining parts in part-based models are modeled mainly as graphical models [41,188,296,297]. The authors of [211] model the human body as a collection of planar patches undergoing affine motion, and soft constraints penalize the distance between the articulation points predicted by adjacent affine models. In a similar approach, authors [212] constrain the body joint displacements to be the same under the affine models of the adjacent parts, resulting in a simple linear constrained least squares optimization for kinematic constrained part tracking.

Motion model parameters can also be directly optimized. For example, the Contracting Curve Density algorithm (CCD) [298] refines an initial parameter set to fit a parametric curve model to an image. Additionally, the Wandering–Stable–Lost (WSL) model [299] was developed in the context of parametric motion estimation. Motion information can also be extracted as flow fields. For example, the articulated flow fields are inferred by using pose-labeled segmentation [300]. Part motion estimation methods are also proposed [213,214,215].

Sampling is another way to solve motion models. The Markov chain Monte Carlo (MCMC) technique is frequently used in motion-based human pose estimation as a sampling method. It samples the complex solution space. The set of solution samples generated by the Markov chain weakly converges to a stationary distribution equivalent to the posterior distribution. Data-driven MCMC framework [177,216] allows the design of good proposal functions derived from image observations such as face, head–shoulder contour, and skin color blobs. Particle Message Passing (PAMPAS) can also be used to solve motion-based problems in the form of non-parametric belief propagation [217,218]. Additionally, a scale checking and adjusting algorithm is proposed to automatically adjust the perspective scales during the tracking process to tackle the multiple perspective scales problem [301].

Gaussian Processes (GP), which can be used to specify distribution over function, are generalizations of Gaussian distributions defined over infinite index sets [259,302,303]. After incorporating temporal information, the Gaussian Process Latent Variable Model (GPLVM) [45,219,220,221,222] is proposed to learn the distributions of styles of human motion with multi-factor correspondence to the latent variables. In addition, the use of Gaussian Process Dynamical Models (GPDMs) [223] have been advocated for learning human pose and motion priors for 3D people tracking [304]. Furthermore, based on learning dynamical models, Gaussian auto regressive processes can be learned by automatically partitioning the parameter space into regions with similar dynamical characteristics [305]. For a particular motion sequence, a circle dynamics model (CDM) is used when the style is assumed constant over time to restrict the content of different styles to lie on the same trajectory [110].

The locality-constrained linear coding (LLC) algorithm [152] is another way to encode motion attributes in reduced dimensions. LLC is performed to learn the nonlinear mapping in order to reconstruct a 3D human pose. A novel motionlet LLC coding is proposed in a discriminative framework using motionlets as codebooks in [151].

## 5. Datasets, Error Measurements, and Toolkits

### 5.1. Datasets

In this section, widely-used validation data sets for human pose estimation are collected and shown in Table 2. We divide the collected data sets into two categories: still images and image sequences, to distinguish between sequential image sequences with temporal coherence between frames and those without. For each data set, the content is listed in the third column: some are action types to which collected poses belong, and others are the compositions of the data set. In the last column, the image numbers included in each data set are displayed. The table displays the collected data sets in approximately chronological order within each category.

### 5.2. Error Measurements

For the validation of human pose estimation algorithms, various error measurements are used. These error measurements can be split into two categories, based on whether human pose is represented as a collection of body parts or body joints. Body part-based error measurements include the PCP (Percentage of Correct Parts) metric [329]) and Mean (over all angles) in [127]. Body joint-based error measurements include PDJ (Percent of Detected Joints) metric, APK (Average Precision of Key Point) [229], and PCK (Probability of Correct Key Point) [229]. In addition, these two error measurement methodologies are combined as an overall measurement [88].

### 5.3. Toolkits

OpenVL provides a high-level interface to image segmentation [330]. Pose detection is a component in this library. It introduces an abstraction layer above the sophisticated techniques in vision: an abstraction layer is developed through which a description of the problem may be provided, rather than requiring the selection of a particular algorithm that is confined to computer vision experts. The algorithm can be chosen by searching in a table [8]. The table contains four algorithms, four image descriptions, seven target descriptions, and three output requirements. Various elements are combined, and users can select a proper algorithm based on descriptions.

## 6. Discussion

Human pose estimation from monocular images has been extensively studied over past decades, and the problem is still far from being completely solved. Different from other computer vision problems, human pose estimation requires the localization of human body parts from images and their assembly based on a predefined human body structure. What is more, it is mostly a regression problem which has a continuous output space. One interesting problem is to model the human pose space or to confine the high-dimensional solution space. For example, instead of using the Euclidean difference of two deformations—which is not capable of providing a meaningful measure of shape dissimilarity—the authors of [144] explore lie bodies, a Riemannian structure which factors body shape deformations into multiple causes or represents shape as a linear combination of basis shapes. In this space, arithmetic over body shape deformations makes sense. Furthermore, when working with deep learning, an extensive collection of human poses would be useful for training deep nets, but this would be tons of work due to the high degree of freedom of human poses and ambiguous human body joint limits.

Until now, almost all solutions are aiming at designing an algorithm, but very few work on algorithm efficiency. To be successfully applied in real-life applications, this is a factor that must be considered. So, the proposal of efficient human pose estimation algorithms for real-time application could provide a bright future to this problem. Efficient and accurate algorithms based on deep learning are still an open challenge. Successful algorithm design and engineering experience are both required for further advancements in this direction. Either an algorithm that can take advantage of various types of data sets could be proposed, or a new large-scale data set should be collected to facilitate the solution.

Another unsolved challenge is partial and self-occlusions. Almost all human pose estimation algorithms use predefined human body structure to be efficient and deterministic; only a few learn the human body structure from the images. How to efficiently and accurately model human body structure from images is still an open challenge.

## Figures and Tables

**Table 1:** A complete overview of human pose estimation from monocular images.

Components	Categories	Sub-Categories	
**Features**	**Low-level Features**	**(1) Shape**: silhouettes [31,32,33,34], contours [35,36], edges [37,38]	
		**(2) Color**: [36,39,40]	
		**(3) Textures**: [41]	
	**Mid-level Features**	**(1) Local features**: like Fourier descriptor [42], shape contexts [43,44,45,46,47], geometric signature [48], Poisson features [49], histogram of oriented gradients (HOG) [50,51,52], relational edge distribution [53], Scale Invariant Feature Transform (SIFT) [54,55] and SIFT-like features [56,57], edgelet features [58], and shapelet features [59]	
		**(2) Global Features**: like object foreground map [46], max-covering [46], dense grid features [50,56,60]	
		**(3) Multilevel hierarchical encodings**: like Hierarchical Model and X (HMAX) [61], hyperfeatures [62] , spatial pyramid [63], vocabulary tree and Multilevel Spatial Blocks (MSB) [64]	
		**(4) Automatic extracted features**: like from a convolutional neural network (CNN) [65,66,67]	
	**High-level Features**	**(1) Context** [8]	
		**(2) Combined body parts** [68,69,70]	
	**Motion Features**	**(1) Optical flow related:** dense optical flow [71], robust optical flow [72]	
		**(2) Combined motion features**: like combined edge energy and motion boundaries [73]	
**Human Body Models**	**Kinematic Models**	**(1) Predefined model**: pictorial structure models (PSM) [71,74], tree-structured models [41,75,76,77,78,79,80,81], improved tree-structured models [36,82,83,84,85,86,87,88,89]	
		**(2) Learned graph structure**: learned pairwise body part relations [90], learned tree structure based on Bayesian Networks [91,92]	
	**Planar**	**Planar model**: Active Shape Model (ASM) [93,94,95,96], cardboard [97]	
	**Volumetric Models**	**(1) Cylindrical model**: [98]	
		**(2) Meshes**: Shape Completion and Animation of People (SCAPE) [99,100,101,102,103], enhanced SCAPE model [104], 3D models with shading [105], and others [94,99,106]	
	**Prior Models**	**Motion prior model**: motion priors from motion capture data [107,108,109,110], joint limits [111], random forests (RFs) and principal direction analysis [2], physics-based models with dynamics [112,113]	
**Methods**	**Generative**	**Generative methods** [22,114,115,116,117,118,119]	
	**Discrimi-native Methods**	** Learning-based Methods**	**(1) Mapping-based methods**: Support Vector Machines (SVMs) [120,121,122], Relevance Vector Machines (RVMs) [32,123,124,125], Mixture of Experts (MoE) [126,127,128], Bayesian Mixtures of Experts (BME) [129,130], direct mapping [32,60,120,121,122,123,124,125,131,132,133], 2D to 3D pose boosting [78,134,135,136,137]; supervised and unsupervised [64,138] and semi-supervised methods [139,140,141]
			**(2) Space Learning**: manifold learning [24,34,142,143,144,145,146,147], subspace learning [148,149], dimensional reduction [150], and others [56,64,151,152]
			**(3) Bag-of-words**: [130]
			**(4) Deep learning**: part detection with the accurate localization of human body parts through deep learning networks [66,153,154], features learned through deep learning and modeling the human body with kinematic graphical models [155,156], learning both with deep learning [90,157,158,159], and enhanced deep learning algorithms [160,161,162,163,164]
		**Examplar**	Randomized trees [165], Random Forests [166,167], and sparse representation [168,169,170,171]
	**Combined Methods**		**Combined Methods of discriminative and generative methods**: [147,172,173,174,175,176,177,178,179,180]
	**Top-Down Methods**		**Top-Down Methods**: [181]
	** Bottom-Up Methods**	**Pixel-based**	Boosting pose estimation accuracy iteratively [97,182,183,184,185,186], pose estimation combined with segmentation [187,188,189,190,191,192,193]
		**Part-based Methods**	**(1) Pictorial Structures**: Pictorial Structures [36,77,79,189,194,195,196,197] and its deformations [79,195,198,199]
			**(2) Enhanced Kinematic Models**: better appearance [187], more modes [88], cascaded models [36,84,200,201], and loopy-graph models [87,202,203]
	** Combined Methods**	**First**	**Combined methods of detection- and recognition-based methods**: [37,60,204,205,206,207]
		**Second**	**Combined methods of pixel-based and part-based methods**: [46,193,205,208,209,210]
	**Motion-based Methods**		Motion model [211,212], kinematic constraints from motion [213,214,215], sampling based tracking [177,216,217,218], Gaussian Process Latent Variable Model (GPLVM) [45,219,220,221,222], Gaussian Process Dynamical Models (GPDMs) [223]

**Table 2:** Publicly available human pose estimation data sets.

Data Set		Content	Image No.
Type	Name		
Still Images	PASCAL VOC 2009	Phoning, Riding Horse, Running, Walking	7054
	Gamesourcing [306]	300 images each from PARSE, BUFFY, LEEDS	48
	Leeds Sports Pose Dataset [307]	Athletics, Badminton, Baseball, Gymnastics, Parkour, Soccer, Tennis, Volleyball	2000
	“We are family” stickmen [308]		
	PASCAL VOC 2012	Ten actions, including jumping, phoning, playing instrument, etc.	11,530
	PASCAL Stickmen [309]		549
	PEAR [310]	Five subjects performing seven predefined	
	KTH Multiview Football Dataset I [311]	2D dataset	5907
	KTH Multiview Football Dataset II [312]	3D dataset	2400
	FLIC (Frames Labeled In Cinema) [313]	Images in 30 movies	5003
	FLIC-full [314]	Images in 30 movies	20,928
	FLIC-plus [315]		
	PARSE [316]	Mostly playing sports	305
	MPII Human Pose Dataset [317]	hockey ice, rope skipping, trampoline, rock climbing, cricket batting, etc.	25,000
	Poses in the Wild [318]		900
	Multi Human Pose [319]		
	Human 3.6H (H36M) [320]	Seventeen scenarios, including discussion, smoking, taking photo, talking on the phone, etc.	3.6 million
	ChaLearn Looking at People 2015: Human Pose Recovery [321]		8000
Image Sequences	CMU-Mocap [322]	Jumping Jacks, Climbing a ladder, Walking	
	Utrecht Multi-Person Motion [323]	Multi-person motion image sequences	
	HumanEva-I [324]	Walk, Jog, Gestures, ThrowCatch, Box	74,267
	HumanEva-II		
	TUM Kitchen [325]		>20,000
	Buffy Pose Classes (BPC) [326]	Episodes 2 to 6 of the 5th season the TV show “Buffy the vampire slayer” (BTVS)	748
	Buffy Stickmen V3.01 [327]	Five episodes of the fifth season of BTVS	
	H3D database	With 3D joint positions	1240
	Video Pose [328]	Forty-four short clips from Buffy the Vampire Slayer, Friends, and LOST	1286
	Video Pose 2.0 dataset		900
